# Intra-Articular Injections into the Inferior versus Superior Compartment of the Temporomandibular Joint: A Systematic Review and Meta-Analysis

**DOI:** 10.3390/jcm12041664

**Published:** 2023-02-19

**Authors:** Maciej Chęciński, Kamila Chęcińska, Natalia Turosz, Maciej Sikora, Dariusz Chlubek

**Affiliations:** 1 Department of Oral Surgery, Preventive Medicine Center, Komorowskiego 12, 30-106 Cracow, Poland; 2 Department of Glass Technology and Amorphous Coatings, Faculty of Materials Science and Ceramics, AGH University of Science and Technology, Mickiewicza 30, 30-059 Cracow, Poland; 3 Institute of Public Health, Jagiellonian University Medical College, Skawińska 8, 31-066 Cracow, Poland; 4 Department of Maxillofacial Surgery, Hospital of the Ministry of Interior, Wojska Polskiego 51, 25-375 Kielce, Poland; 5 Department of Biochemistry and Medical Chemistry, Pomeranian Medical University, Powstańców Wielkopolskich 72, 70-111 Szczecin, Poland

**Keywords:** temporomandibular joint, inferior TMJ compartment, discomandibular space, temporomandibular disorders, intra-articular injections, hyaluronic acid, arthrocentesis, hypertonic dextrose

## Abstract

This systematic review and meta-analysis aimed to validate the hypothesis that intra-articular injections into the inferior temporomandibular joint compartment are more efficient than analogous superior compartment interventions. Publications reporting differences between the above-mentioned techniques in the domains of revealing articular pain, decreasing the Helkimo index, and abolishing mandibular mobility limitation were included. Medical databases covered by the Bielefeld Academic Search Engine, Google Scholar, PubMed, ResearchGate, and Scopus engines were searched. The risk of bias was assessed using dedicated Cochrane tools (RoB2, ROBINS-I). The results were visualized with tables, charts, and a funnel plot. Six reports describing five studies with a total of 342 patients were identified. Of these, four trials on a total of 337 patients were qualified for quantitative synthesis. Each eligible report was at moderate risk of bias. From 19% to 51% improvement in articular pain, 12–20% lower Helkimo index, and 5–17% higher maximum mouth opening were observed. The evidence was limited by the small number of eligible studies, discrepancies regarding the substances used, possible biases, and the differences in observation times and scheduled follow-up visits. Despite the above, the advantage of inferior over superior compartment temporomandibular joint intra-articular injections is unequivocal and encourages further research in this direction.

## 1. Introduction

### 1.1. Background

**Figure 1:**
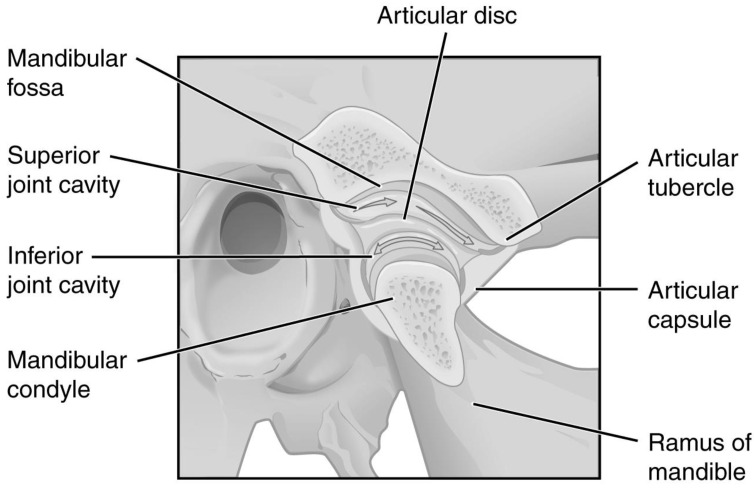
Temporomandibular joint. Modified. OpenStax College, CC BY 3.0 (creativecommons.org/licenses/by/3.0).

The temporomandibular joints (TMJs) are located symmetrically on both sides of the head. Properly functioning, they allow the teeth to move relative to each other, thus biting and chewing [1,2]. The articular disc divides each TMJ cavity into superior (discotemporal) and inferior (discomandibular) compartments, which dictates a complicated pattern of possible movements of the articular head relative to the acetabulum. (Figure 1) [3,4]. Articular disc displacement with or without reduction is referred to as TMJ internal derangement [5]. This dysfunction causes disc clicking, hence inflammation manifested by articular pain and reduced mandibular mobility, resulting in deterioration in patient-reported quality of life [6,7]. The severity of the above, along with imaging tests, allows for classifying the internal derangement stage [8,9,10,11,12]. Depending on the severity of the temporomandibular disorders, treatment regimens consist of pharmacotherapy, physiotherapy, occlusal rearrangement (including splint therapy), intra-muscular injections, intra-articular injections, and arthroplasty [13,14,15,16,17,18,19,20].

Efficient in immediate ailment relief, intra-articular injections also provide satisfactory effects in several months of observation [21,22]. They allow for TMJ cavity rinsing (arthrocentesis) and the administration of autogenous preparations (blood derivatives and cell transplants) or drugs [6,23,24,25,26,27]. Arthrocentesis can be used as a stand-alone technique or precede the injection of a selected substance [23,28,29]. The protocols of drug administration with or without prior lavage differ from each other in the injectable used [21,30,31,32,33,34]. In the group of pharmaceuticals, hyaluronic acid (HA), corticosteroids, hypertonic dextrose, and anesthetics are applicable [30,31,32,33,34,35]. The significance of the type of injectable used is still debated, prompting the assessment of the importance of other factors, such as the specific injection site [27,28,36].

The specific intra-articular location of depositing the injected substance is presumed to be determinative due to the different motor functions of individual TMJ compartments [37,38]. The full assessment of the complexity of these movements is still pending, but it is assumed that hinge or rotational movements take place below the articular disc, and translation or gliding movements above it [37,38,39]. Mastication capacity, resulting from a combination of the movements described above, translates into an overall assessment of the quality of life [7,40]. Based on subdiscal arthroscopy, the role of the inferior compartment for the proper functioning of the TMJ and the hitherto underestimated role of abnormalities below the articular disc in the etiopathogenesis of internal disarrangement are regarded to be greater than previously thought [39].

This all leads to careful selection of the rinsed TMJ compartment and the exact place of injectable deposition [41,42]. The superior compartment, bounded superiorly by the articular fossa on the temporal bone, is wider (approximately 1.2 mL) and, therefore, easier to access for a needle or an endoscope [12,37,38,39,43]. Puncturing into the inferior compartment partially surrounding the head of the mandible is technically more difficult due to the shape of this part of the joint cavity and its smaller volume (approximately 0.9 mL) [37,38,39,42]. Precise injection poses a challenge for clinicians due to the blind nature of this intra-articular intervention type [44,45,46,47]. This problem is being addressed with ever-improved puncture protocols and various imaging techniques (radiography, ultrasonography, and magnetic resonance imaging) [41,44,45,46,47,48,49,50].

### 1.2. Rationale

The more significant role of the inferior TMJ compartment for the initial phase of mouth opening encourages the consideration of inferior compartment intra-articular injections as potentially more efficient in relieving articular pain, reducing the overall severity of internal disarrangement expressed by the Helkimo index, and improving mandibular mobility [37,38,39,40,42]. The comparison of the effects of inferior or both compartments versus superior intra-articular injections was the subject of a systematic review with meta-analysis published in 2012 by Li et al. [42]. A greater improvement in both articular pain and maximum mouth opening domains with the interventions studied compared to standard upper compartment administrations was observed [42,49,50,51,52]. Nevertheless, both the meta-analysis result itself and later published comments argued for the need to support the initial conclusions with further clinical trials [42,53,54]. Heterogeneity in terms of technique (lower or both spaces) and more than a decade since publication further justify the need to determine the current state of knowledge about the validity of intra-articular injections for specific TMJ compartments [42].

### 1.3. Objective

This meta-analysis aims to validate the hypothesis that intra-articular injections into the inferior temporomandibular joint compartment are more efficient in relieving articular pain and abolishing mouth-opening limitation than analogous superior compartment interventions.

## 2. Materials and Methods

### 2.1. Eligibility Criteria

Eligibility was determined according to the PICO methodology, specifying inclusion and exclusion criteria for the problem, intervention, comparison, and outcomes (Table 1) [55,56,57]. Any types of publications containing data from original clinical trials were included without any time frame limit. The problem studied was the diagnosis of temporomandibular joint internal derangement in the Wilkes classification stages II to V [11]. Studies in which patients underwent TMJ inferior compartment arthrocentesis and/or intra-articular injections of self-derived preparations (e.g., PRP, PRGF, I-PRF, MSC) or drugs (e.g., hyaluronan, corticosteroids, hypertonic dextrose, anesthetics) and their combinations were qualified. Treatment involving arthroscopy or open joint surgery within the same procedure was rejected. Studies that did not include the treatment group for the sole inferior TMJ compartment only were also excluded. This decision was motivated by the inability to estimate the effectiveness of the individual components of a combined injection into both TMJ compartments [42,51,52]. As a reference, the same type of intervention in terms of substance, dosage regimen, and duration of treatment within the superior joint compartment was required. Comparisons conducted by injections into both joint spaces were excluded for the reasons mentioned above [42,51,52]. The quantitative evaluation of the effectiveness of therapy in the domains of articular pain severity, Helkimo index, and the range of mandibular mobility was taken into account [58,59]. Subjective pain assessment on a visual analog scale (VAS) or numeric rating scale (NRS) was accepted [19,59]. The range of mandibular mobility expressed as abduction, protrusive movement, and lateral movements were allowed, respecting this hierarchy. Papers reporting at least one of the outcomes mentioned above were accepted.

### 2.2. Information Sources

The Bielefeld Academic Search Engine (BASE), ClinicalTrials.gov, Google Scholar, PubMed, ResearchGate, and Scopus search engines were used to identify potentially eligible reports throughout medical databases [60]. All final searches were performed on 1 November 2022. Additionally, the references of each eligible publication were searched for further records.

### 2.3. Search Strategy

The following search strategy was used:

(inferior OR lower) AND (superior OR upper) AND (compartment OR space) AND temporomandibular AND (joint OR articulation) AND (arthrocentesis OR rinsing OR lavage OR injection OR administration OR viscosupplementation).

Individual search queries for each search engine are shown in Table A1, Appendix A.

### 2.4. Selection Process

Reports were selected in accordance with the Preferred Reporting Items for Systematic Reviews and Meta-Analyses (PRISMA) protocol using the Rayyan tool [61,62,63]. The convergence of blind assessments of two judges (M.C. and K.C.) was expressed by the value of Cohen’s kappa coefficient. Reports considered potentially eligible by any of the judges during screening were promoted to the full-text assessment stage. A reference search of the included studies was conducted for further potentially eligible items.

### 2.5. Data Collection Process

Data were extracted from the content of reports without the use of automation tools. In the case of discrepancies between the values collected by two independent reviewers (M.C. and N.T.), joint verification was performed, and the decision was made through discussion.

### 2.6. Data Items

The following data items were collected to identify individual studies and to characterize the test and control groups: (1) first author of the report; (2) publication year; (3) study type; (4) diagnosis; (5) study and control group sizes; (6) sex and age structures; (7) injectable and dosage; (8) eligible outcome domains; (9) follow-up time. The study group was considered to be patients receiving injections into the inferior TMJ compartment, and the study group consisted of individuals injected within the superior TMJ compartment.

For the purposes of the synthesis, the values of the following variables were extracted from the study reports: (1) the intensity of articular pain; (2) Helkimo index (HI); (3) maximum mouth opening (MMO) [19,58,59]. The values of these variables before treatment initiation (initial), at intermediate visits, and after treatment completion (final) were collected for the study and control groups.

### 2.7. Study Risk of Bias Assessment

The risk of bias within the studies was assessed using the revised Cochrane risk-of-bias tool for randomized trials (RoB2) and the tool for assessing the risk of bias in non-randomized studies of interventions (ROBINS-I) for randomized and non-randomized trials, respectively [64,65]. In the case of reports covering a larger number of patient groups or other interventions, only the data useful in this meta-analysis were considered in assessing the risk of bias.

### 2.8. Effect Measures

To assess the efficiency of inferior versus superior compartment TMJ treatment in pain, Helkimo Index, and MMO domains, the percentage decrease in the corresponding variable values was compared each time in the study and control groups according to the formula:

e = e_i_ − e_s_ = v_if_ ÷ v_i0_ × 100% − v_sf_ ÷ v_s0_ × 100%,

where e_i_ and es are the treatment efficiencies in the inferior and superior TMJ compartment groups, respectively, and v stands for final (v_if_, v_sf_) and initial (v_i0_, v_s0_) variable values, with an analogous compartment designation.

### 2.9. Synthesis Methods

Studies with no greater than a moderate risk of bias were allowed for syntheses. In the absence of information on the values of the necessary variables, a given study was not taken into account in a given synthesis. Pain severity in VAS or NRS was converted proportionally to values in the range 0–10, with only the VAS values used when using both scales in one study [19,59]. With the different methods of measuring the extent of the mandibular abduction within one study (e.g., maximum mouth opening, maximum unassisted opening, maximum pain-free opening, etc.), only the one with the highest values was used for synthesis. For data visualization purposes, it was assumed that a month consists of 4 weeks. The results of individual studies were presented in tabular form, and the results of syntheses were presented graphically in charts. The exploration of possible reasons for the heterogeneity of the studies was carried out using the meta-regression method. Each of the syntheses was carried out under the condition of completeness of data in a given domain, which excluded the need to assess the risk of reporting bias. For all assessments, a significance level of 0.05 was adopted. For the meta-assessment of the publication bias presence, the results were visualized in a funnel plot.

## 3. Results

### 3.1. Study Selection

**Figure 2:**
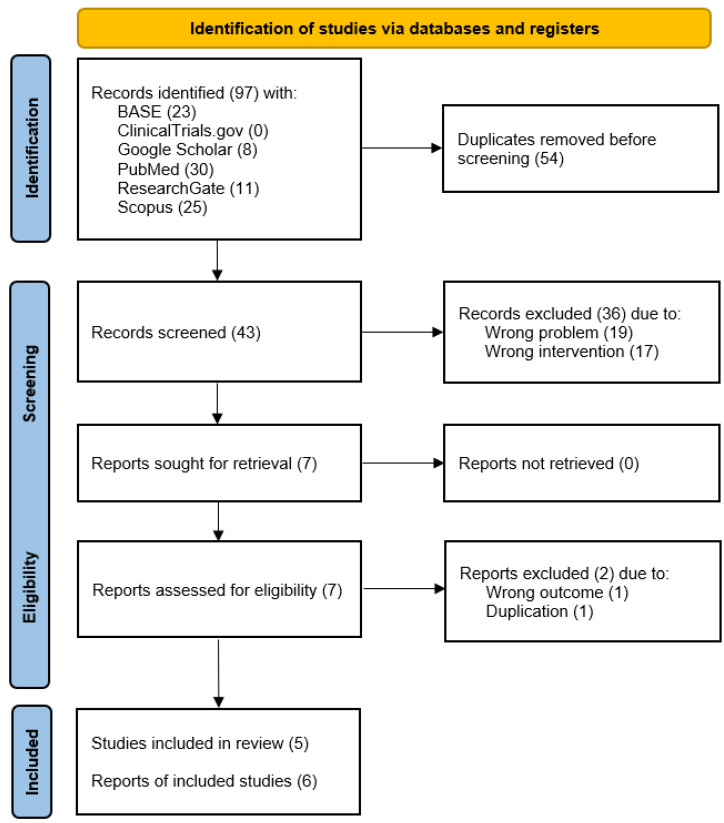
PRISMA flow diagram.

Searching with six engines led to the identification of 97 records, of which 54 duplicates indicated by the Rayyan tool were manually removed [61,62]. Thus, 43 entries qualified for the screening stage and assessment of the titles’ and abstracts’ content, resulting in the exclusion of a further 36 records inconsistent with the problem or intervention sought. The compliance of abstract qualifications according to two judges, expressed by Cohen’s kappa coefficient, was 0.91. Seven reports qualified for full-text evaluation, three of which described the same trial by Long [50,66]. Among them, a record with errors in the title and author list fields pointing to the same full text as the correct one was excluded [50]. The same was done with a conference abstract from 2008 with consistent characteristics of patient groups as the scientific article from 2009 [50,66]. However, the formal reason for rejecting the latter was the lack of numerical values of the variables in any of the eligible outcome domains. One of the articles qualifying for the synthesis was a systematic review containing the required data from two studies meeting the inclusion criteria [42,49,50]. The report from the first one was found in the course of this selection process, regardless of its inclusion in the review paper [50]. The second study was originally published in the Chinese-language journal “Guoji kouqiang yixue zazhi”, from which the source report was retrieved and included in the synthesis [49]. Ultimately, the meta-analysis was based on six reports describing five eligible studies comparing the effectiveness of injection therapies to the lower versus the upper TMJ compartment [41,42,48,49,50,67]. These studies were conducted on a total of 342 patients [41,48,49,50,67]. The reference search yielded no further results. The main steps of the selection process were visualized as a PRISMA-compliant diagram (Figure 2) [68].

### 3.2. Study Characteristics

All qualifying studies were conducted on the basis of groups diagnosed with TMJs internal derangement. Only in the study by Fouda et al. did the stage of the disease in patients allow for the reduction of the displaced disc; in others, the blockage was irreducible. The size of the study and control groups ranged from 1 to 73 subjects, which was taken into account in the meta-analysis. The numerous missing data did not allow for precise age determination and, in particular, the age differentiation between the study and control groups. In general, the interventions consisted of intra-articular injection of 1 to 2 mL of fluid, although the substances used differed depending on the study design. The number of administrations ranged from 1 to 4, and in the case of several interventions, the intervals between them were 1–2 weeks. The amplitude of mandibular abduction was determined in four out of five studies, and both articular pain and Helkimo index values were presented in two out of five reports. The maximum observation time for at least some members of the study group ranged from 2–3 days to 9 months. The characteristics of the individual studies are presented in Table 2.

### 3.3. Risk of Bias in Studies

The overall risk of bias for reporting changes in pain, Helkimo’s index, and MMO domains for the patient groups receiving lower and upper TMJ compartment injections was moderate in all included studies but one (Table 3). The report by Ozawa et al. was included in the review due to an excerpt of the results that were considered eligible according to the PICOS criteria. Concerns about the selectively included data from this paper led to an assessment of the high risk of bias, thus excluding the study from syntheses.

### 3.4. Results of Individual Studies

#### 3.4.1. Articular Pain

**Figure 3:**
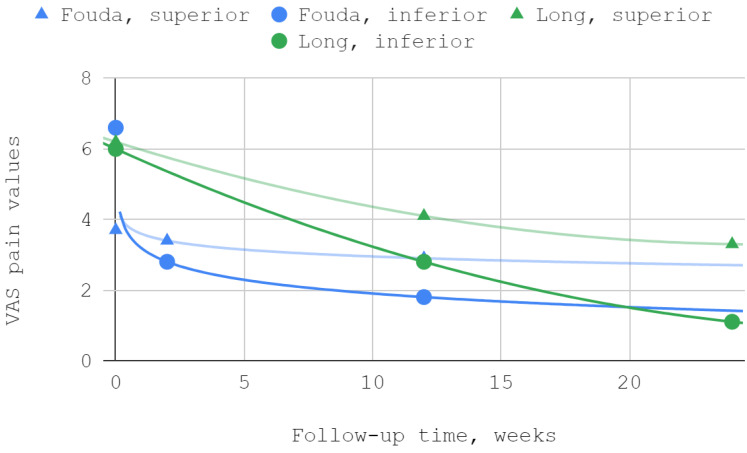
Articular pain in VAS over time.

In both studies that included articular pain in the VAS as a separate variable, improvement was observed for both superior and inferior TMJ injections. The best fits were obtained in the study by Fouda et al. for logarithmic curves, and in the report by Long et al., trends followed second-degree polynomials. The differences between the results in the study group and the control group were statistically significant in the reports of both teams of authors (Table 4, Figure 3).

#### 3.4.2. Helkimo Index

**Figure 4:**
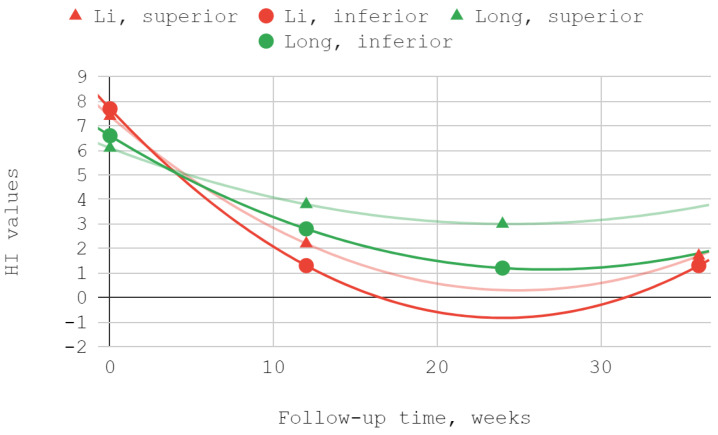
Helkimo index (HI) over time.

The severity of TMJ dysfunction expressed by the Helkimo index subsided as a result of treatment for both superior and inferior compartment injections. A good fit was obtained in each series using a second-degree polynomial trend line. Efficiency differences in favor of the group injected in the lower part of the joint were statistically significant during both follow-up visits in the course of observation of the team of Li et al. In a study by Long et al. numerically greater differences were observed, but statistically significant only on the second of the two follow-up visits (Table 5, Figure 4).

#### 3.4.3. Maximum Mouth Opening

**Figure 5:**
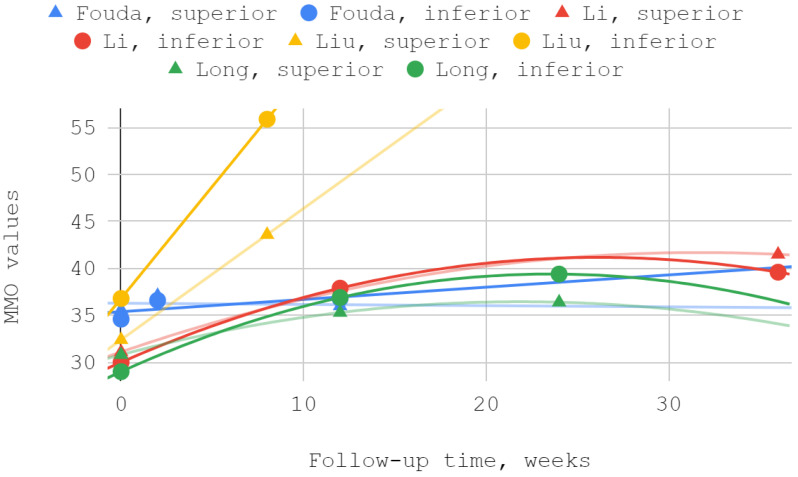
Maximum mouth opening (MMO) over time—whole chart.

**Figure 6:**
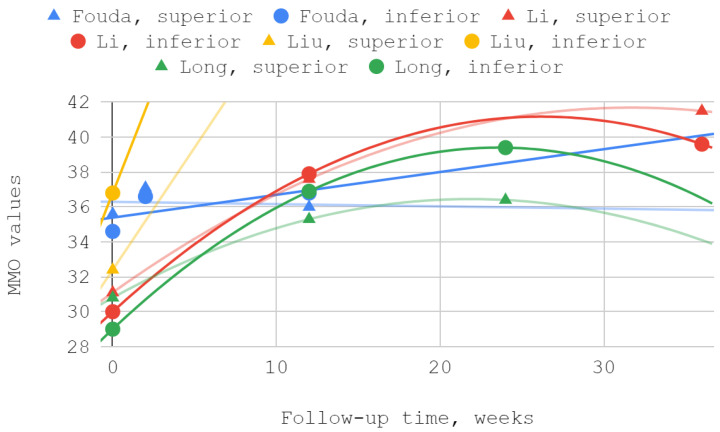
Maximum mouth opening (MMO) over time—chart fragment close-up.

The mobility of the mandible, expressed by the values of its abduction, was measured in each of the discussed reports. Injection treatment was effective in this domain each time, regardless of the injected TMJ compartment. The results of Ozawa et al. for acute cases are numerically presented but not illustrated due to the small size of the patient groups. MMO values from reports by Li et al. and Long et al. showed a second-degree polynomial trend. The other series differed from the pattern of standard (linear, polynomial, logarithmic) fits. For a general view, linear trend lines have been used in these cases. Of the seven measurements during the follow-up periods described in various reports, three differences between the inferior versus superior groups were not statistically significant, including the only difference in favor of the superior group (Table 6, Figure 5 and Figure 6).

### 3.5. Results of Syntheses

**Figure 7:**
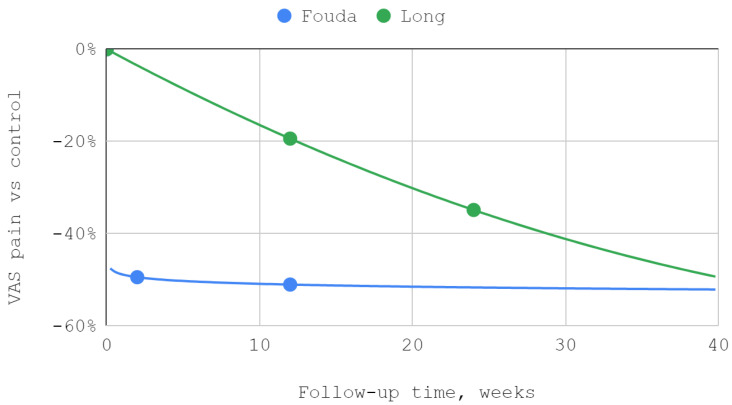
The efficiency of inferior versus superior compartment TMJ treatment in VAS articular pain domain over time.

**Figure 8:**
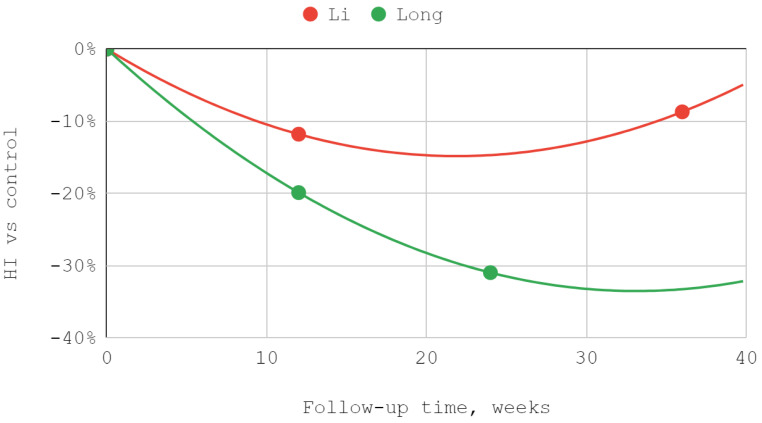
The efficiency of inferior versus superior compartment TMJ treatment in Helkimo index (HI) domain over time.

**Figure 9:**
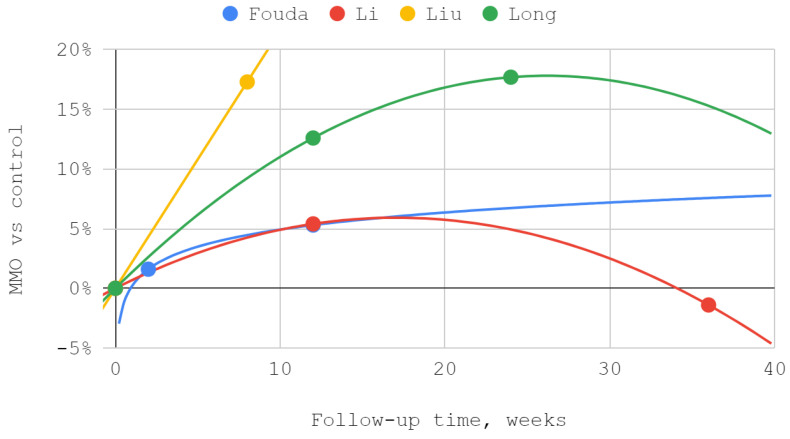
The efficiency of inferior versus superior compartment TMJ treatment in maximum mouth opening (MMO) domain over time.

**Figure 10:**
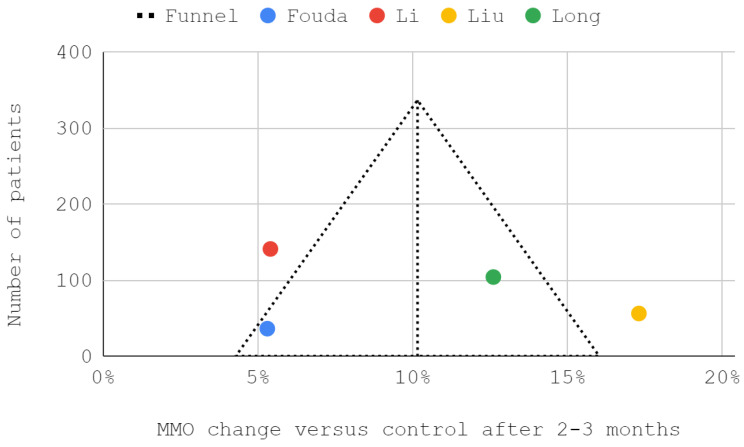
A funnel plot of the efficiency of inferior versus superior compartment TMJ treatment in maximum mouth opening (MMO) domain after 2–3 months.

The effectiveness of lower compartment treatment compared to control varied significantly between studies. Articular pain difference in the report of Long et al. increased gradually, in contrast to the high constant difference observed after treatment by Fouda et al. The pattern of differences in the effectiveness of both techniques suggests a possible loss of superiority of injection into the lower compartment over time in relation to the Helkimo index. However, during the reported follow-up period, this superiority was still present. The results achieved in the study groups relative to the control regarding MMO suggest an increasing advantage of treatment oriented to the lower TMJ compartment. Nevertheless, it can be suspected that this advantage peaks between 3 and 9 months of observation and decreases further. For all but one study, second-degree polynomial fits of inferior versus superior compartment efficiencies were found to be the most appropriate. Exceptionally, for Fouda et al.’s results, the natural-based logarithmic trendlines were presented as the most accurate fits. The difference in efficiencies between superior and inferior compartment TMJ injections after 2–3 months was assessed in each of the synthesized studies. Therefore, this data series was used to illustrate the convergence of results presented in individual reports. The outline of the funnel on the horizontal axis is the mean difference in performance minus and increased by the standard deviation. The height of the funnel on the vertical axis is determined by the total number of 337 patients treated with injections into the upper and lower TMJ compartments. The outlier outside the funnel contour coexists with outlying trend curves in the MMO domain for the study by Liu et al. Despite the datapoint outlier in the funnel plot, the result of Li et al. is consistent with that of Fouda et al. (Figure 7, Figure 8, Figure 9 and Figure 10).

## 4. Discussion

### 4.1. Interpretation of the Results

In all qualifying studies, a greater improvement in VAS articular pain, HI, and MMO domains occurred due to inferior than superior compartment TMJ intra-articular injections [41,48,49,50,67]. However, the lack of homogeneity in terms of the injectables used throughout the analyzed studies must be emphasized [41,48,49,50,67]. The reports by Li et al. and Long et al. showed similar trends for HI and MMO rates [41,49]. The improvement in MMO was higher for injections into the inferior compartment of TMJ than into the superior compartment by 5.4% in the report of Li et al., and by 12.6% in the study of Long et al. [41,49]. Regression analysis showed that the MMO improvements progressed over time in smaller and smaller increments [41,49]. The described trends did not differ from the results of other studies observed for viscosupplementation of HA [21,41,49,69,70]. In both analyzed studies, patients received intra-articularly equal doses of 1 mL HA [41,49]. The decrease in the Helkimo index after 3 months was 11.8% higher for injections into the inferior compartment of the TMJ than for the superior compartment in the Li et al. study and 19.9% higher in the Long et al. study [41,49]. Both Li at al. and Long et al. administered 1 mL of HA intra-articularly using a congruent technique [41,49]. However, Li et al. administered one dose more, which may explain the better results obtained [41,49].

Of note are the results of Liu et al., who also reported a significant increase in MMO administering HA to the TMJ space [49]. After two months, MMO increased by 51.9% for injections into the inferior compartment of TMJ, while it increased by 34.6% for the superior compartment [49]. In the course of the discussed trial, apart from the injections, the patients also underwent splint therapy [49]. In a recent systematic review, the first administration of HA was proven to be more effective than subsequent administrations [21]. These partially explain the outlier result of single HA administration preceded by arthrocentesis and combined with splint therapy in the study by Liu et al. [21,24,49,71].

Fouda et al. noted the lowest increase in MMO (1.1–6.4%) among analyzed studies [48]. This may be due to the diagnosis of disk displacement with reduction, in contrast to the other reports where disk displacement without reduction was treated [41,48,49,50,67]. The weaker results in the report of Fouda et al. may be also explained by the administration of 25% hypertonic dextrose (after receiving 2% mepivacaine locally) in contrast to the HA used in other trials [41,48,49,50]. Recent systematic reviews showed that the use of HA results in a greater increase in MMO and often greater improvement in VAS than administering hypertonic dextrose [21,28]. According to these data, in patients who received HA, the final values of pain were from 14 to 62% of the initial intensity [21]. The reduction in joint pain levels among patients treated with dextrose ranged from 33 to 76% of initial pain [28]. In the report of Fouda et al., articular pain on the VAS scale was also measured, yielding better results within the inferior compartment of TMJ where a 72.7% decrease in pain was noted (51.1% for the superior TMJ compartment) [48]. These values fit into the range specified in the review mentioned above [28]. The results of the Fouda et al. trial show that in both domains, improvement occurred immediately after the intervention, with no significant gain in effectiveness over follow-up [48]. This is consistent with the results of other prolotherapy reports [28,48,72,73,74]. In the material collected in this review, it was observed that the longer the follow-up time, the greater the improvement in MMO [41,48,49,50]. Li et al. and Long et al. monitored patients for 3 to 6 months longer than Fouda et al., which makes it possible that the highest MMO values for the prolotherapy group were not recorded [41,48,50].

### 4.2. Injection Technique

Making sure that the drug is administered to the correct compartment of the temporomandibular joint is crucial for taking advantage of this aspect [41,44,45,46,47,48,49,50,67]. Ahlqvist et al. used an X-ray source under the patient’s head to correctly locate the bevel of the needle tip [45]. The bevel should be positioned next to the condyle facing its surface in case of inferior compartment injections, and below the posterior slope of the articular tubercle when administering to the upper compartment [45]. Yeung et al. showed the possibility of using an intraoperative navigation system (Stryker Leibinger, Freiburg, Germany) which, based on magnetic resonance imaging (MRI) of the TMJ, enables injection into the correct compartment of the joint [46]. Recent studies presented the use of ultrasonography when performing intra-articular TMJ injections [44,47]. Cha et al. reported that ultrasound-guided injections, especially into the lower compartment, are performed with significantly higher accuracy than those based solely on anatomical landmarks [44]. Januzzi et al. additionally used cone beam computed tomography (CBCT) to confront the precise location of osseous structures with facial access points [47]. The lack of universal acceptance of a specific method of puncture into the inferior TMJ compartment inspires further anatomical and clinical research in this direction [44,45,46,47]. Injecting both compartments as part of one intervention, preferably with a single injection, seems to be a desirable solution [18,51,52].

### 4.3. Limitations

A similar systematic review was published more than a decade ago by Li et al. [42]. These authors then identified two studies comparing inferior compartment injections and another two comparing both-compartment injections to upper compartment TMJ injections [42,49,50,51,52]. On the basis of the meta-analysis, the superiority of both techniques over administrations to the upper TMJ compartment was demonstrated [42,49,50,51,52]. Limitations of the work of Li et al. resulting from the small number of heterogeneous source studies are still a current problem [42]. Despite no mixing of inferior compartment injection with both-compartment injection studies within the systematic review reported here, the heterogeneity resulting from the variety of administered substances remained [41,48,49,50,67]. Literature reviews indicate that the type of substance used may have a significant impact on the effectiveness of injection therapy [27,28,29,75]. Therefore, the small number of studies identified, discrepancies regarding the substances used and intra-articular injection techniques, uncertainties regarding possible biases, and the differences in observation times and scheduled follow-up visits between individual trials are the limitations of the evidence included in this review [41,48,49,50].

## 5. Conclusions

The advantage of inferior over superior compartment intra-articular injections within the temporomandibular joint is unequivocal. However, the evidence is limited to four studies (337 patients in total), each with a moderate risk of bias. It can be conservatively assumed that after 2–3 months of follow-up, the numerical benefits of the lower compartment intra-articular injections are close to the ranges shown in this meta-analysis. These are from 19% to 51% improvement in articular pain, from 12% to 20% lower Helkimo index, and from 5% to 17% higher maximum mouth opening compared to supradiscal interventions. The inhomogeneity of the analyzed studies in terms of the substances used does not allow for the determination of average efficiency gain due to the application of the technique in question. Further clinical trials followed by a meta-analysis taking into account subgroups depending on the injectables seem to be justified.

## Figures and Tables

**Table 1:** Summarized eligibility criteria.

	Inclusion	Exclusion
Problem	TMJ internal derangement	Animal studies
Intervention	Arthrocentesis, intra-articular injection, or a combination thereof within the inferior TMJ compartment	Atroscopy or open joint surgery as part of the same procedure
Comparison	Same intervention for the superior TMJ compartment	Intervention in both TMJ compartments in one patient group
Outcomes	Articular pain, Helkimo index, mandibular mobility	-

TMJ—temporomandibular joint.

**Table 2:** Study characteristics.

First Author	Publication Year	Study Type	Diagnosis	Group Sizes	Age, Mean	Intervention	Number of Instances, Interval	Eligible Outcomes	Follow-Up Visits
Fouda [48]	2018	RT	DDwR	18—inferior, 18—superior	In range 18–42, N/S	1.5 mL of 25% hypertonic dextrose + 0.2 mL anesthetic	4, 1 week	Pain, MMO	2 weeks and 3 months
Li [41]	2014	RT	DDwoR	68—inferior, 73—superior	N/S, in range 31.4–34.1	1 mL HA	4, 2 weeks	MMO, HI	3 and 9 months
Liu [49]	2010	RT	DDwoR	28—inferior, 28—superior	14–48, 25.7	Arthrocentesis + 1–2 mL HA + 2 months of splint therapy	1, N/A	MMO	2 months
Long [50]	2009	RT	DDwoR	54—inferior, 50—superior	N/S, in range 25.6–30.6	1 mL anesthetic + 1 mL HA	3, 2 weeks	Pain, HI, MMO	3 and 6 months
Ozawa [67]	1996	nRT	DDwoR	4—inferior, 1—superior	17–37, 22.2	Pumping arthrocentesis	1, N/A	MMO	2–3 days

RT—randomized trial; nRT—non-randomized trial; DDwR—disk displacement with reduction; DDwoR—disk displacement without reduction; N/S—not specified; HA—Hyaluronic Acid; N/A—not applicable; MMO—maximum mouth opening; HI—Helkimo Index.

**Table 3:** Risk of bias assessment.

First Author	Randomization Process	Confounding	Selection of Participants in the Study	Classification of Interventions	Deviations from Intended Interventions	Missing Data	Measurement of Outcomes	Selection of the Reported Result	Overall Bias
Fouda [48]	Low	N/A	N/A	N/A	Low	Low	Unclear	Low	Moderate
Li [41]	Unclear	N/A	N/A	N/A	Low	Moderate	Unclear	Low	Moderate
Liu [49]	Unclear	N/A	N/A	N/A	Low	Low	Moderate	Low	Moderate
Long [50]	Low	N/A	N/A	N/A	Low	Low	Unclear	Low	Moderate
Ozawa [67]	N/A	High	High	Low	Low	Low	Unclear	Low	High

Columns 2–9 list the domains for which the bias was assessed. N/A—not applicable.

**Table 4:** Articular pain.

First Author	TMJ Compartment	Initial Pain	Pain after 2 Weeks	Pain after 3 Months	Pain after 6 Months
Fouda [48]	Superior	3.7 ± 2.7 (100%; 18 pts)	3.4 ± 3.0 (91.9%; 18 pts)	2.9 ± 3.1 (78.4%; 18 pts)	N/S
	Inferior	6.6 ± 2.5 (100%; 18 pts)	2.8 ± 2.8 (42.4%; 18 pts)	1.8 ±2.1 (27.3%; 18 pts)	N/S
	Difference	0% (*p* < 0.05)	−49.5% (*p* < 0.05)	−51.1% (*p* < 0.05)	N/S
Long [50]	Superior	6.2 ± 0.2 (100%; 50 pts)	N/S	4.1 ± 1.9 (66.1%; 50 pts)	3.3 ± 2.3 (53.2%; 50 pts)
	Inferior	6.0 ± 0.2 (100%; 54 pts)	N/S	2.8 ± 1.7 (46.7%; 54 pts)	1.1 ± 1.3 (18.3%; 54 pts)
	Difference	0% (*p* > 0.05)	N/S	−19.4% (*p* < 0.05)	−34.9% (*p* < 0.05)

TMJ—temporomandibular joint; pain—articular pain in 0–10 VAS; pts—patients; N/S—not specified.

**Table 5:** Helkimo index.

First Author	TMJ Compartment	Initial Helkimo Index	Helkimo Index after 3 Months	Helkimo Index after 6 Months	Helkimo Index after 9 Months
Li [41]	Superior	7.4 ± 3.1 (100%; 73 pts)	2.2 ± 2.0 (29.6%; 65 pts)	N/S	1.7 ± 2.2 (24.5%; 44 pts)
	Inferior	7.7 ± 3.3 (100%; 68 pts)	1.3 ± 1.4 (17.8%; 61 pts)	N/S	1.3 ± 1.9 (15.8%; 30 pts)
	Difference	0% (*p* > 0.05)	−11.8% (*p* < 0.05)	N/S	−8.7% (*p* < 0.05)
Long [50]	Superior	6.1 ± 3.3 (100%; 50 pts)	3.8 ± 2.8 (62.3%; 50 pts)	3.0 ± 2.4 (49.2%; 50 pts)	N/S
	Inferior	6.6 ± 2.3 (100%; 54 pts)	2.8 ± 2.0 (42.4%; 54 pts)	1.2 ± 1.5 (18.2%; 54 pts)	N/S
	Difference	0% (*p* > 0.05)	−19.9% (*p* > 0.05)	−31.0% (*p* < 0.05)	N/S

TMJ—temporomandibular joint; pts—patients; N/S—not specified.

**Table 6:** Maximum mouth opening.

First Author	TMJ Compartment	Initial MMO	MMO after 2–3 Days	MMO after 2 Weeks	MMO after 2 Months	MMO after 3 Months	MMO after 6 Months	MMO after 9 Months
Fouda [48]	Superior	35.6 ± 5.5 (100%; 18 pts)	N/S	37.1 ± 4.4 (104.2%; 18 pts)	N/S	36.0 ± 4.2 (101.1%; 18 pts)	N/S	N/S
	Inferior	34.6 ± 2.4 (100%; 18 pts)	N/S	36.6 ± 1.4 (105.8%; 18 pts)	N/S	36.8 ± 1.2 (106.4%; 18 pts)	N/S	N/S
	Difference	0% (*p* > 0.05)	N/S	1.6% (*p* < 0.05)	N/S	5.3% (*p* < 0.05)	N/S	N/S
Li [41]	Superior	31.1 ± 7.9 (100%; 73 pts)	N/S	N/S	N/S	37.6 ± 6.5 (120.9%; 65 pts)	N/S	41.5 ± 6.4 (133.4%; 44 pts)
	Inferior	30.0 ± 6.8 (100%; 68 pts)	N/S	N/S	N/S	37.9 ± 5.9 (126.3%; 61 pts)	N/S	39.6 ± 5.8 (132.0%; 30 pts)
	Difference	0% (*p* > 0.05)	N/S	N/S	N/S	5.4% (*p* > 0.05)	N/S	−1.4% (*p* > 0.05)
Liu [49]	Superior	32.4 ± 2.3 (100%, 28 pts)	N/S	N/S	43.6 ± 5.1 (134.6%; 28 pts)	N/S	N/S	N/S
	Inferior	36.8 ± 1.4 (100%; 28 pts)	N/S	N/S	55.9 ± 2.9 (151.9%; 28 pts)	N/S	N/S	N/S
	Difference	0% (*p* > 0.05)	N/S	N/S	17.3% (*p* < 0.05)	N/S	N/S	N/S
Long [50]	Superior	30.8 ± 4.9 (100%; 50 pts)	N/S	N/S	N/S	35.3 ± 4.7 (114.6%; 50 pts)	36.4 ± 5.0 (118.2%; 50 pts)	N/S
	Inferior	29.0 ± 4.7 (100%; 54 pts)	N/S	N/S	N/S	36.9 ± 4.6 (127.2%; 54 pts)	39.4 ± 4.4 (135.9%; 54 pts)	N/S
	Difference	0% (*p* > 0.05)	N/S	N/S	N/S	12.6% (*p* > 0.05)	17.7% (*p* < 0.05)	N/S
Ozawa [67]	Superior	34.0 ± 0.0 (100%, 1 pts)	40.0 (117.6%; 1 pts)	N/S	N/S	N/S	N/S	N/S
	Inferior	22.5 ± 4.3 (100%; 4 pts)	39.3 ± 2.9 (174.7%; 4 pts)	N/S	N/S	N/S	N/S	N/S
	Difference	0% (N/A)	57.1% (N/A)	N/S	N/S	N/S	N/S	N/S

TMJ—temporomandibular joint; MMO—maximum mouth opening in millimeters; pts—patients; N/S—not specified; N/A—not applicable.

## Data Availability

The protocol of this systematic review is available in the Prospective Register of Systematic Reviews (PROSPERO) under number CRD42022381667. All collected data are available within the body of this article.

## Appendix A

**Table A1:** Search queries.

Search Engine	Search Query
BASE	(inferior OR lower) AND (superior OR upper) AND (compartment OR space) AND temporomandibular AND (joint OR articulation) AND (arthrocentesis OR rinsing OR lavage OR injection OR administration OR viscosupplementation)
ClinicalTrials.gov	(arthrocentesis OR rinsing OR lavage OR injection OR administration OR viscosupplementation) | (inferior OR lower) AND (superior OR upper) AND (compartment OR space) AND temporomandibular AND (joint OR articulation)
Google Scholar	allintitle: (inferior OR lower) AND (superior OR upper) AND (compartment OR space) AND temporomandibular AND (joint OR articulation) AND (arthrocentesis OR rinsing OR lavage OR injection OR administration OR viscosupplementation)
PubMed	(inferior OR lower) AND (superior OR upper) AND (compartment OR space) AND temporomandibular AND (joint OR articulation) AND (arthrocentesis OR rinsing OR lavage OR injection OR administration OR viscosupplementation)
ResearchGate	(inferior OR lower) AND (superior OR upper) AND (compartment OR space) AND temporomandibular AND (joint OR articulation) AND (arthrocentesis OR rinsing OR lavage OR injection OR administration OR viscosupplementation)
Scopus	TITLE-ABS-KEY ((inferior OR lower) AND (superior OR upper) AND (compartment OR space) AND temporomandibular AND (joint OR articulation) AND (arthrocentesis OR rinsing OR lavage OR injection OR administration OR viscosupplementation))

## References

[1] Speksnijder C.M., Mutsaers N.E.A., Walji S. (2022). Functioning of the Masticatory System in Patients with an Alloplastic Total Temporomandibular Joint Prostheses Compared with Healthy Individuals: A Pilot Study. Life.

[2] Tobe S., Ishiyama H., Nishiyama A., Miyazono K., Kimura H., Fueki K. (2022). Effects of Jaw-Opening Exercises with/without Pain for Temporomandibular Disorders: A Pilot Randomized Controlled Trial. Int. J. Environ. Res. Public Health.

[3] Habibi H.A., Ozturk M., Caliskan E., Turan M. (2022). Quantitative assessment of temporomandibular disc and masseter muscle with shear wave elastography. Oral Radiol..

[4] Walker T.F., Broadwell B.K., Noujeim M.E. (2017). MRI assessment of temporomandibular disc position among various mandibular positions: A pilot study. CRANIO^®^.

[5] Yildiz S., Balel Y., Tumer M.K. (2022). Evaluation of prevalence of temporomandibular disorders based on DC/TMD Axis I diagnosis in Turkish population and correlation with Axis II. J. Stomatol. Oral Maxillofac. Surg..

[6] Sikora M., Sielski M., Chęciński M., Nowak Z., Czerwińska-Niezabitowska B., Chlubek D. (2022). Repeated Intra-Articular Administration of Platelet-Rich Plasma (PRP) in Temporomandibular Disorders: A Clinical Case Series. J. Clin. Med..

[7] Bitiniene D., Zamaliauskiene R., Kubilius R., Leketas M., Gailius T., Smirnovaite K. (2018). Quality of life in patients with temporomandibular disorders. A systematic review. Stomatologija.

[8] Schiffman E., Ohrbach R., Truelove E., Look J., Anderson G., Goulet J.-P., List T., Svensson P., Gonzalez Y., Lobbezoo F. (2014). Diagnostic Criteria for Temporomandibular Disorders (DC/TMD) for Clinical and Research Applications: Recommendations of the International RDC/TMD Consortium Network* and Orofacial Pain Special Interest Group†. J. Oral Facial Pain Headache.

[9] Pigg M., Law A., Nixdorf D., Renton T., Sharav Y., Svensson P., Ernberg M., Peck C., Alstergren P., Kaspo G. (2020). International Classification of Orofacial Pain, 1st edition (ICOP). Cephalalgia.

[10] Iwaszenko S., Munk J., Baron S., Smoliński A. (2021). New Method for Analysis of the Temporomandibular Joint Using Cone Beam Computed Tomography. Sensors.

[11] Smolka W., Yanai C., Smolka K., Iizuka T. (2008). Efficiency of arthroscopic lysis and lavage for internal derangement of the temporomandibular joint correlated with Wilkes classification. Oral Surg. Oral Med. Oral Pathol. Oral Radiol. Endodontol..

[12] Machoy M., Szyszka-Sommerfeld L., Rahnama M., Koprowski R., Wilczyński S., Woźniak K. (2020). Diagnosis of Temporomandibular Disorders Using Thermovision Imaging. Pain Res. Manag..

[13] Turosz N., Chęcińska K., Chęciński M., Kamińska M., Nowak Z., Sikora M., Chlubek D. (2022). A Scoping Review of the Use of Pioglitazone in the Treatment of Temporo-Mandibular Joint Arthritis. Int. J. Environ. Res. Public Health.

[14] Nitecka-Buchta A., Walczynska-Dragon K., Kempa W.M., Baron S. (2019). Platelet-Rich Plasma Intramuscular Injections—Antinociceptive Therapy in Myofascial Pain Within Masseter Muscles in Temporomandibular Disorders Patients: A Pilot Study. Front. Neurol..

[15] Nowak Z., Chęciński M., Nitecka-Buchta A., Bulanda S., Ilczuk-Rypuła D., Postek-Stefańska L., Baron S. (2021). Intramuscular Injections and Dry Needling within Masticatory Muscles in Management of Myofascial Pain. Systematic Review of Clinical Trials. Int. J. Environ. Res. Public Health.

[16] Kulesa-Mrowiecka M., Piech J., Gaździk T.S. (2021). The Effectiveness of Physical Therapy in Patients with Generalized Joint Hypermobility and Concurrent Temporomandibular Disorders—A Cross-Sectional Study. J. Clin. Med..

[17] Dowgierd K., Pokrowiecki R., Kulesa Mrowiecka M., Dowgierd M., Woś J., Szymor P., Kozakiewicz M., Lipowicz A., Roman M., Myśliwiec A. (2022). Protocol for Multi-Stage Treatment of Temporomandibular Joint Ankylosis in Children and Adolescents. J. Clin. Med..

[18] Sun H., Su Y., Song N., Li C., Shi Z., Li L. (2018). Clinical Outcome of Sodium Hyaluronate Injection into the Superior and Inferior Joint Space for Osteoarthritis of the Temporomandibular Joint Evaluated by Cone-Beam Computed Tomography: A Retrospective Study of 51 Patients and 56 Joints. Med. Sci. Monit..

[19] Byra J., Kulesa-Mrowiecka M., Pihut M. (2020). Physiotherapy in hypomobility of temporomandibular joints. Folia Med. Cracov..

[20] Sikora M., Chęciński M., Nowak Z., Chlubek D. (2021). Variants and Modifications of the Retroauricular Approach Using in Temporomandibular Joint Surgery: A Systematic Review. J. Clin. Med..

[21] Chęciński M., Sikora M., Chęcińska K., Nowak Z., Chlubek D. (2022). The Administration of Hyaluronic Acid into the Temporomandibular Joints’ Cavities Increases the Mandible’s Mobility: A Systematic Review and Meta-Analysis. J. Clin. Med..

[22] Chung P.-Y., Lin M.-T., Chang H.-P. (2019). Effectiveness of platelet-rich plasma injection in patients with temporomandibular joint osteoarthritis: A systematic review and meta-analysis of randomized controlled trials. Oral Surg. Oral Med. Oral Pathol. Oral Radiol..

[23] Guarda-Nardini L., De Almeida A., Manfredini D. (2021). Arthrocentesis of the Temporomandibular Joint: Systematic Review and Clinical Implications of Research Findings. J. Oral Facial Pain Headache.

[24] Şentürk M.F., Yazıcı T., Gülşen U. (2017). Techniques and modifications for TMJ arthrocentesis: A literature review. CRANIO^®^.

[25] Manafikhi M., Ataya J., Heshmeh O. (2022). Evaluation of the efficacy of platelet rich fibrin (I-PRF) intra-articular injections in the management of internal derangements of temporomandibular joints—A controlled preliminary prospective clinical study. BMC Musculoskelet. Disord..

[26] Chęciński M., Chęcińska K., Turosz N., Kamińska M., Nowak Z., Sikora M., Chlubek D. (2022). Autologous Stem Cells Transplants in the Treatment of Temporomandibular Joints Disorders: A Systematic Review and Meta-Analysis of Clinical Trials. Cells.

[27] Derwich M., Mitus-Kenig M., Pawlowska E. (2021). Mechanisms of Action and Efficacy of Hyaluronic Acid, Corticosteroids and Platelet-Rich Plasma in the Treatment of Temporomandibular Joint Osteoarthritis—A Systematic Review. Int. J. Mol. Sci..

[28] Chęciński M., Chęcińska K., Nowak Z., Sikora M., Chlubek D. (2022). Treatment of Mandibular Hypomobility by Injections into the Temporomandibular Joints: A Systematic Review of the Substances Used. J. Clin. Med..

[29] Cömert Kiliç S., Güngörmüş M., Sümbüllü M.A. (2015). Is Arthrocentesis Plus Platelet-Rich Plasma Superior to Arthrocentesis Alone in the Treatment of Temporomandibular Joint Osteoarthritis? A Randomized Clinical Trial. J. Oral Maxillofac. Surg..

[30] Sikora M., Czerwińska-Niezabitowska B., Chęciński M.A., Sielski M., Chlubek D. (2020). Short-Term Effects of Intra-Articular Hyaluronic Acid Administration in Patients with Temporomandibular Joint Disorders. J. Clin. Med..

[31] Liapaki A., Thamm J.R., Ha S., Monteiro J.L.G.C., McCain J.P., Troulis M.J., Guastaldi F.P.S. (2021). Is there a difference in treatment effect of different intra-articular drugs for temporomandibular joint osteoarthritis? A systematic review of randomized controlled trials. Int. J. Oral Maxillofac. Surg..

[32] Sit R.W.-S., Reeves K.D., Zhong C.C., Wong C.H.L., Wang B., Chung V.C., Wong S.Y., Rabago D. (2021). Efficacy of hypertonic dextrose injection (prolotherapy) in temporomandibular joint dysfunction: A systematic review and meta-analysis. Sci. Rep..

[33] Sipahi A., Satilmis T., Basa S. (2015). Comparative study in patients with symptomatic internal derangements of the temporomandibular joint: Analgesic outcomes of arthrocentesis with or without intra-articular morphine and tramadol. Br. J. Oral Maxillofac. Surg..

[34] Yapici-Yavuz G., Simsek-Kaya G., Ogul H. (2018). A comparison of the effects of Methylprednisolone Acetate, Sodium Hyaluronate and Tenoxicam in the treatment of non-reducing disc displacement of the temporomandibular joint. Med. Oral.

[35] Kałużyński K., Trybek G., Smektała T., Masiuk M., Myśliwiec L., Sporniak-Tutak K. (2016). Effect of methylprednisolone, hyaluronic acid and pioglitazone on histological remodeling of temporomandibular joint cartilage in rabbits affected by drug-induced osteoarthritis. Postep. Hig. Med. Dosw (Online).

[36] Xie Y., Zhao K., Ye G., Yao X., Yu M., Ouyang H. (2022). Effectiveness of Intra-Articular Injections of Sodium Hyaluronate, Corticosteroids, Platelet-Rich Plasma on Temporomandibular Joint Osteoarthritis: A Systematic Review and Network Meta-Analysis of Randomized Controlled Trials. J. Evid.-Based Dent. Pract..

[37] Whyte A., Boeddinghaus R., Bartley A., Vijeyaendra R. (2021). Imaging of the temporomandibular joint. Clin. Radiol..

[38] Alomar X., Medrano J., Cabratosa J., Clavero J.A., Lorente M., Serra I., Monill J.M., Salvador A. (2007). Anatomy of the Temporomandibular Joint. Semin. Ultrasound CT MRI.

[39] González-García R., Moreno-Sánchez M., Moreno-García C., Román-Romero L., Monje F. (2018). Arthroscopy of the Inferior Compartment of the Temporomandibular Joint: A New Perspective. J. Maxillofac. Oral Surg..

[40] Sikora M., Sielski M., Chęciński M., Chęcińska K., Czerwińska-Niezabitowska B., Chlubek D. (2022). Patient-Reported Quality of Life versus Physical Examination in Treating Temporomandibular Disorders with Intra-Articular Platelet-Rich Plasma Injections: An Open-Label Clinical Trial. Int. J. Environ. Res. Public Health.

[41] Li C., Long X., Deng M., Li J., Cai H., Meng Q. (2015). Osteoarthritic Changes After Superior and Inferior Joint Space Injection of Hyaluronic Acid for the Treatment of Temporomandibular Joint Osteoarthritis with Anterior Disc Displacement Without Reduction: A Cone-Beam Computed Tomographic Evaluation. J. Oral Maxillofac. Surg..

[42] Li C., Zhang Y., Lv J., Shi Z. (2012). Inferior or Double Joint Spaces Injection Versus Superior Joint Space Injection for Temporomandibular Disorders: A Systematic Review and Meta-Analysis. J. Oral Maxillofac. Surg..

[43] Pihut M., Szuta M., Ferendiuk E., Zeńczak-Więckiewicz D. (2014). Evaluation of Pain Regression in Patients with Temporomandibular Dysfunction Treated by Intra-Articular Platelet-Rich Plasma Injections: A Preliminary Report. BioMed Res. Int..

[44] Cha Y.H., Park J.K., Yang H.M., Kim S.H. (2019). Ultrasound-guided versus blind temporomandibular joint injections: A pilot cadaveric evaluation. Int. J. Oral Maxillofac. Surg..

[45] Ahlqvist J., Legrell P.E. (1993). A technique for the accurate administration of corticosteroids in the temporomandibular joint. Dentomaxillofac. Radiol..

[46] Yeung R.W.K., Xia J.J., Samman N. (2006). Image-Guided Minimally Invasive Surgical Access to the Temporomandibular Joint: A Preliminary Report. J. Oral Maxillofac. Surg..

[47] Januzzi E., Cunha T.C.A., Silva G., Souza B.D.M., Duarte A.S.B., Zanini M.R.S., Andrade A.M., Pedrosa A.R., Custódio A.L.N., Castro M.A.A. (2022). Viscosupplementation in the upper and lower compartments of the temporomandibular joint checked by ultrasonography in an ex vivo and in vivo study. Sci. Rep..

[48] Fouda A.A. (2018). Change of site of intra-articular injection of hypertonic dextrose resulted in different effects of treatment. Br. J. Oral Maxillofac. Surg..

[49] Liu J.J., Mu H., Zhang D.S., Long X., Wang Q.H., Fu X.L. (2010). Lower joint cavity treatment of temporomandibular joint with anterior disc displacement without reduction. Int. J. Stomatol..

[50] Long X., Chen G., an Cheng A.H., Cheng Y., Deng M., Cai H., Meng Q. (2009). A Randomized Controlled Trial of Superior and Inferior Temporomandibular Joint Space Injection with Hyaluronic Acid in Treatment of Anterior Disc Displacement Without Reduction. J. Oral Maxillofac. Surg..

[51] Cheng X.L. (2005). An outcome analysis of two methods of intra-capsular injection of prednisolone for temporomandibular disorders. Chin. J. Prim. Med. Pharm..

[52] Liu C.M., Shi Z.D., Yi X.Z., Guo C.Z., Zhang X. (2003). Observation of the therapeutic effects on TMD by injection HA into both superior and inferior temporomandibular joint cavities. J. Dent. Prev. Treat.

[53] Ebrahim S. (2012). Methodological Limitations of a Systematic Review Evaluating Inferior or Double Joint Spaces Injection Versus Superior Joint Space Injection for Temporomandibular Disorders. J. Oral Maxillofac. Surg..

[54] Li C., Shi Z. (2012). Reply to Dr Shanil Ebrahim on Inferior or Double Joint Spaces Injection Versus Superior Joint Space Injection for Temporomandibular Disorders: A Systematic Review and Meta-Analysis. J. Oral Maxillofac. Surg..

[55] Turfah A., Liu H., Stewart L.A., Kang T., Weng C., Otero P., Scott P., Martin S.Z., Huesing E. (2022). Extending PICO with Observation Normalization for Evidence Computing. Studies in Health Technology and Informatics.

[56] Methley A.M., Campbell S., Chew-Graham C., McNally R., Cheraghi-Sohi S. (2014). PICO, PICOS and SPIDER: A comparison study of specificity and sensitivity in three search tools for qualitative systematic reviews. BMC Heal. Serv. Res..

[57] Eriksen M.B., Frandsen T.F. (2018). The impact of patient, intervention, comparison, outcome (PICO) as a search strategy tool on literature search quality: A systematic review. J. Med Libr. Assoc..

[58] Leamari V.M., de Rodrigues A.F., Camino Junior R., Luz J.G.C. (2019). Correlations between the Helkimo indices and the maximal mandibular excursion capacities of patients with temporomandibular joint disorders. J. Bodyw. Mov. Ther..

[59] Emshoff R., Emshoff I., Bertram S. (2010). Estimation of clinically important change for visual analog scales measuring chronic temporomandibular disorder pain. J. Orofac. Pain.

[60] Gusenbauer M. (2022). Search where you will find most: Comparing the disciplinary coverage of 56 bibliographic databases. Scientometrics.

[61] Valizadeh A., Moassefi M., Nakhostin-Ansari A., Hosseini Asl S.H., Saghab Torbati M., Aghajani R., Maleki Ghorbani Z., Faghani S. (2022). Abstract screening using the automated tool Rayyan: Results of effectiveness in three diagnostic test accuracy systematic reviews. BMC Med. Res. Methodol..

[62] Ouzzani M., Hammady H., Fedorowicz Z., Elmagarmid A. (2016). Rayyan—A web and mobile app for systematic reviews. Syst. Rev..

[63] Guimarães N.S., Ferreira A.J., Silva R.D.C.R., de Paula A.A., Lisboa C.S., Magno L., Ichiara M.Y., Barreto M.L. (2022). Deduplicating records in systematic reviews: There are free, accurate automated ways to do so. J. Clin. Epidemiology.

[64] Sterne J.A.C., Savović J., Page M.J., Elbers R.G., Blencowe N.S., Boutron I., Cates C.J., Cheng H.-Y., Corbett M.S., Eldridge S.M. (2019). RoB 2: A revised tool for assessing risk of bias in randomised trials. BMJ.

[65] Sterne J.A., Hernán M.A., Reeves B.C., Savović J., Berkman N.D., Viswanathan M., Henry D., Altman D.G., Ansari M.T., Boutron I. (2016). ROBINS-I: A tool for assessing risk of bias in non-randomised studies of interventions. BMJ.

[66] Long X. (2008). Comparative Study of Inferior Versus Superior Joint Space Injection of Sodium Hyaluronate in Patients with Anterior Disc Displacement Without Reduction of the Temporomandibular Joint: A Randomized Controlled Trial. J. Oral Maxillofac. Surg..

[67] Ozawa M., Okaue M., Kaneko K., Hasegawa M., Matsunaga S., Matsumoto M., Hori M., Kudo I., Takagi M. (1996). Clinical Assessment of the Pumping Technique in Treating TMJ Arthrosis with Closed Lock. J. Nihon Univ. Sch. Dent..

[68] Page M.J., McKenzie J.E., Bossuyt P.M., Boutron I., Hoffmann T.C., Mulrow C.D., Shamseer L., Tetzlaff J.M., Akl E.A., Brennan S.E. (2021). The PRISMA 2020 statement: An updated guideline for reporting systematic reviews. BMJ.

[69] Yang W., Liu W., Miao C., Sun H., Li L., Li C. (2018). Oral Glucosamine Hydrochloride Combined with Hyaluronate Sodium Intra-Articular Injection for Temporomandibular Joint Osteoarthritis: A Double-Blind Randomized Controlled Trial. J. Oral Maxillofac. Surg..

[70] Fonseca R.M.D.F.B., Januzzi E., Ferreira L.A., Grossmann E., Carvalho A.C.P., de Oliveira P.G., Vieira É.L.M., Teixeira A.L., Almeida-Leite C.M. (2018). Effectiveness of Sequential Viscosupplementation in Temporomandibular Joint Internal Derangements and Symptomatology: A Case Series. Pain Res. Manag..

[71] Wiechens B., Paschereit S., Hampe T., Wassmann T., Gersdorff N., Bürgers R. (2022). Changes in Maximum Mandibular Mobility Due to Splint Therapy in Patients with Temporomandibular Disorders. Healthcare.

[72] Zarate M.A., Frusso R.D., Reeves K.D., Cheng A.-L., Rabago D. (2020). Dextrose Prolotherapy Versus Lidocaine Injection for Temporomandibular Dysfunction: A Pragmatic Randomized Controlled Trial. J. Altern. Complement. Med..

[73] Louw W.F., Reeves K.D., Lam S.K.H., Cheng A.-L., Rabago D. (2019). Treatment of Temporomandibular Dysfunction with Hypertonic Dextrose Injection (Prolotherapy): A Randomized Controlled Trial with Long-term Partial Crossover. Mayo Clin. Proc..

[74] Lam S.K.H., Reeves K.D., Rabago D. (2016). Dextrose Prolotherapy for Chronic Temporomandibular Pain and Dysfunction: Results of a Pilot-Level Randomized Controlled Study. Arch. Phys. Med. Rehabil..

[75] Zotti F., Albanese M., Rodella L.F., Nocini P.F. (2019). Platelet-Rich Plasma in Treatment of Temporomandibular Joint Dysfunctions: Narrative Review. Int. J. Mol. Sci..

